# Supplementary material for: Identification, Characterization, and Homology Analysis of a Novel Strain of the Crimean–Congo Hemorrhagic Fever Virus from Yunnan, China
Source: Microorganisms. 2024 Jul 19;12(7):1466. doi: 10.3390/microorganisms12071466 (PMC11278756; doi:10.3390/microorganisms12071466)
Supplement: Supplementary file 1 [file microorganisms-12-01466-s001.zip › microorganisms-3075339-supplementary.pdf]

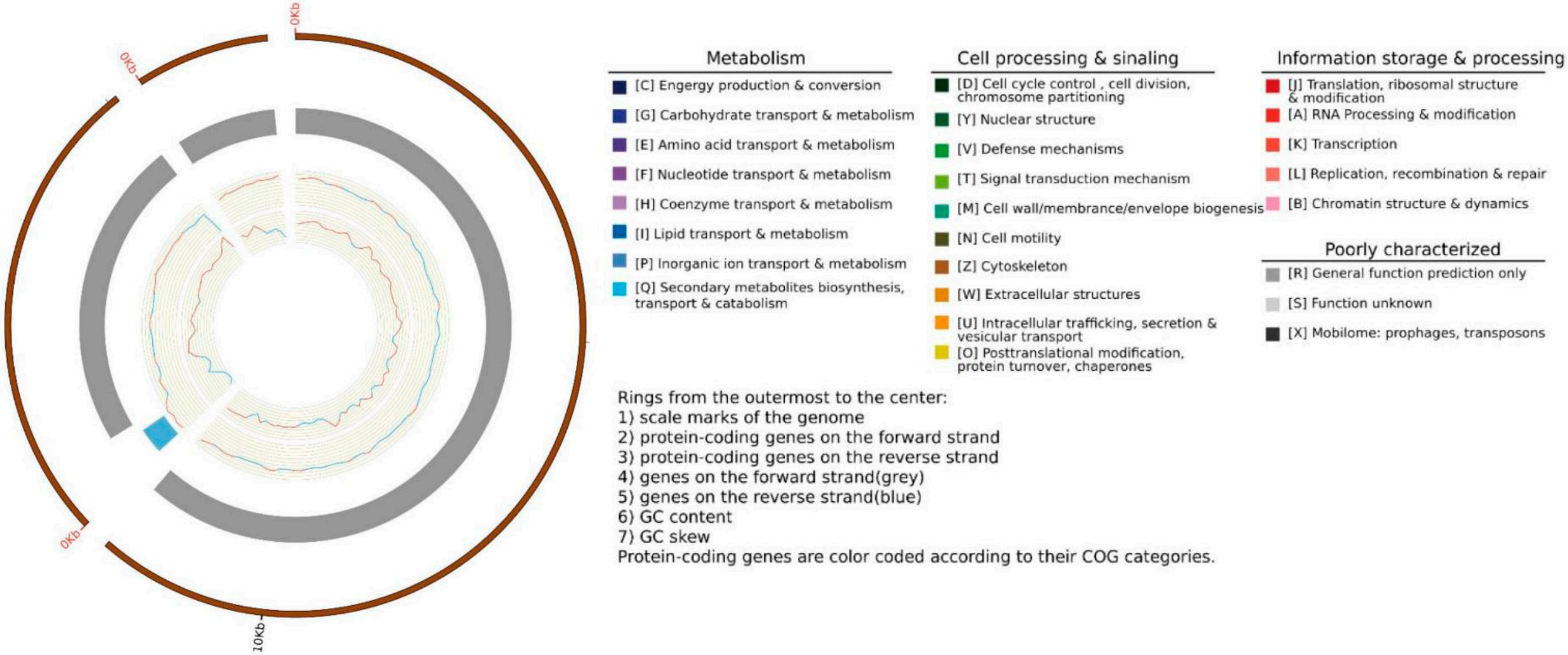

Supplementation Figure Legend

Supplementation Figure S1. CCHFV genome map comprises L, M and S segments. The three discontinuous lines in the genome map's outermost circle represent the L, M, and S segments. 0 kb represents the start site of the gene (ATG), and the L segment is labeled at the 10 kb position, and those less than 10 kb are not labeled.
